# Early skeletal muscle loss and clinical outcomes in critically ill patients in the medical intensive care unit: A retrospective cohort study

**DOI:** 10.1371/journal.pone.0338315

**Published:** 2025-12-18

**Authors:** Soyun Kim, Da Hyun Kang, Dukki Kim, Soyoung Ahn, Mi Ra Lee, Song I Lee

**Affiliations:** 1 Division of Pulmonary and Critical Care Medicine, Department of Internal Medicine, Chungnam National University School of Medicine, Chungnam National University Hospital, Daejeon, Republic of Korea; 2 Department of Rehabilitation Medicine, Chungnam National University Hospital, Chungnam National University School of Medicine, Daejeon, Republic of Korea; 3 Division of Industrial Mathematics, Data Analytics Team, National Institute for Mathematical Sciences, Daejeon, Republic of Korea; Lorestan University, IRAN, ISLAMIC REPUBLIC OF

## Abstract

**Background:**

Acute skeletal muscle wasting is a common complication among critically ill patients and contributes to prolonged stays in the intensive care unit (ICU) and poor outcomes. Although bedside ultrasound of the rectus femoris cross-sectional area (RFcsa) is useful for assessing muscle loss, data on its incidence and predictors in medical ICU populations are limited. Our study aimed to determine the incidence of significant muscle wasting (≥10% reduction in RFcsa) and to identify modifiable predictors, particularly early rehabilitation.

**Methods:**

This retrospective cohort study was conducted at a tertiary academic hospital in South Korea. Seventy-six adult ICU patients who underwent serial ultrasound assessments of RFcsa within 48 h of admission and again on day 7 were included. Patients were categorized based on whether they experienced a ≥ 10% reduction in RFcsa. Clinical characteristics, interventions, and outcomes were compared between groups. Multivariate logistic regression identified predictors of significant muscle loss, ICU mortality, and in-hospital mortality.

**Results:**

A significant reduction in RFcsa (≥10%) was observed in 53.9% of patients (n = 41/76). These patients had longer ICU stays (median 15.0 vs. 10.0 d, p = 0.001) and were less likely to receive Level 3 or higher ICU-level rehabilitation (4.9% vs. 20.0%, p = 0.042). Multivariate analysis revealed that Level 3 or higher rehabilitation was independently associated with reduced muscle wasting (OR: 0.183; 95% CI: 0.035–0.970; p = 0.046). Multivariable analyses revealed that increased total bilirubin and decreased albumin levels were associated with ICU mortality. In-hospital mortality, however, was independently associated with a higher Charlson comorbidity index, elevated bilirubin and C-reactive protein levels, and low albumin. A decrease in RFcsa of at least 10% was not significantly associated with either outcome.

**Conclusions:**

More than half of the patients in the ICU experienced significant muscle loss within the first week of being admitted. Receiving structured rehabilitation at Level 3 or higher was independently associated with reduced muscle wasting. This suggests that early mobilization strategies may help preserve muscle mass in critically ill patients.

## Introduction

Critically ill patients frequently experience substantial loss of skeletal muscle mass during their stay in the intensive care unit (ICU), particularly during the early stages of critical illness [1,2]. This condition, commonly referred to as acute muscle wasting [3], arises from a combination of decreased physical activity, systemic inflammation, increased proteolysis, and impaired protein synthesis. Importantly, rapid muscle wasting is not merely a physiological response; it carries significant clinical consequences [3], contributing to ICU-acquired weakness, prolonged mechanical ventilation [4], extended ICU length of stay [4,5], and increased mortality [6]. Furthermore, many survivors of critical illness suffer from persistent fatigue and physical dysfunction, severely affecting their quality of life after discharge [7,8].

Identifying patients at high risk of muscle wasting remains a clinical challenge. Traditional methods for assessing muscle mass [9,10]—such as computed tomography, magnetic resonance imaging, dual-energy X-ray absorptiometry, and manual muscle testing—are often impractical in the ICU due to patient instability, lack of cooperation, or the need for specialized equipment and transport. In recent years, bedside ultrasound [11,12] has emerged as a feasible and non-invasive tool for real-time quantification of muscle mass in critically ill patients.

Among the various ultrasound-based metrics, the rectus femoris cross-sectional area (RFcsa) has been proposed as a reliable surrogate marker of muscle strength and functional capacity. A reduction in RFcsa of 10% or more upon ICU admission is considered clinically significant, as it correlates with functional decline and poorer outcomes [1,13,14]. Recent study [15] has further validated the clinical utility of muscle ultrasound measurements in predicting ICU-acquired weakness. Decreased muscle cross-sectional area has been associated with prolonged ICU stays and worse functional outcomes.

However, the frequency of muscle loss and its associated risk factors are poorly documented, especially in medical ICU populations. This study aimed to (1) estimate the incidence of significant muscle wasting (≥10% reduction in RFcsa) in critically ill medical ICU patients and (2) identify modifiable clinical predictors of muscle loss, with particular focus on early rehabilitation. We hypothesized that higher levels of early rehabilitation would be associated with reduced muscle wasting and provide insight to support early mobilization strategies in critically ill patients.

## Materials and methods

### Study design and ethical approval

This retrospective cohort study was conducted in the Medical ICU at Chungnam National University Hospital, a tertiary academic center in Daejeon, South Korea, from April 2022 to December 2023.

The study protocol was approved by the Institutional Review Board of Chungnam National University Hospital (IRB No. 2025-05-026). Due to the retrospective nature of the analysis and the use of fully de-identified data, the IRB waived the informed consent requirement.

The clinical data analyzed in this study originated from a quality improvement initiative supported by the Patient-Centered Clinical Research Coordinating Center (PACEN, grant number HC19C0226) and funded by the Ministry of Health & Welfare of the Republic of Korea.

### Patient population and eligibility criteria

Adult patients (aged ≥18 years) admitted to the ICU during this period were eligible for inclusion if they underwent bedside ultrasound assessments of the RFcsa. Attending physicians selectively performed these ultrasound measurements for clinical monitoring of muscle status, not as part of routine ICU care or a predefined research protocol.

Patients were excluded if they died before the follow-up RFcsa measurement, were discharged from the ICU within seven days of admission or had incomplete or invalid ultrasound data due to patient refusal or technical difficulties.

### Variables and outcome measures

Demographic information—including age, sex, height, weight, and body mass index—along with comorbidities, laboratory results, and clinical severity scores (Acute Physiology and Chronic Health Evaluation [APACHE] II and Sequential Organ Failure Assessment [SOFA]) were collected at ICU admission. Frailty status was assessed using the Clinical Frailty Scale, and the risk of sarcopenia was evaluated using the Strength, Assistance with walking, Rising from a chair, Climbing stairs, and Falls (SARC-F) questionnaire.

Ultrasound measurements of the RFcsa were taken with a 7.5-MHz linear-array transducer positioned perpendicular to the long axis of the thigh, two-thirds of the way between the anterior superior iliac spine and the superior patellar border. Measurements were obtained on Day 1 (within 24 hours of ICU admission) and repeated on Day 7 (±1 day) or prior to ICU discharge, following a standardized protocol. The average of three measurements was used for analysis. Muscle loss was defined as the percentage change in RFcsa from baseline to follow-up.

A ≥ 10% reduction in RFcsa was used to define clinically significant muscle wasting. Although this cutoff has not been universally validated, it has been applied in previous studies. Puthucheary et al. [16] observed early RFcsa losses exceeding 10% in critically ill patients. Zhang et al. [17] found that a 12% reduction provided diagnostic value for ICU-acquired weakness. Hrdy et al. [1] used the ≥ 10% threshold and found that 59.6% of their cohort met this criterion. Together, these findings support the use of the ≥ 10% threshold as a clinically meaningful benchmark in ICU populations.

Level 3 ICU rehabilitation and higher refers to progressive, active physical therapy interventions ranging from bedside sitting to ambulation training. These levels include bedside sitting (Level 3), standing training (Level 4), and ambulation training (Level 5). Specific activities include bedside sitting, lower extremity anti-gravity exercises, active upper extremity range-of-motion exercises, resistance band training, sit-to-stand exercises, standing balance practice, chair transfers, strength training, marching in place, walking, and squatting.

Patients receiving Level 3 or higher rehabilitation undergo active therapy for at least 30 minutes per day. In contrast, those not receiving Level 3 or higher rehabilitation receive passive range-of-motion exercises for up to 10 minutes per day. Standard nursing care practices, including scheduled position changes every eight hours, were implemented equally across both groups.

Additional clinical data collected included the use of mechanical ventilation, renal replacement therapy, and length of ICU stay. Outcomes recorded were ICU and hospital mortality.

All data were extracted from the hospital’s electronic medical record system and entered into a standardized case report form for analysis. Patients were classified into two groups based on the degree of RFcsa reduction: those with a decrease of ≥10% and those with a decrease of <10%. This classification was used in subsequent statistical analyses.

### Ultrasound measurement protocol

To ensure consistency and minimize operator variability, two trained examiners underwent standardized muscle ultrasound training three times a week for 1 year before the study began. All measurements were performed using the same ultrasound machine (VIVID GE E UltraEdition with a 6.5 MHz linear transducer [GE L3-12-D]; GE Healthcare, Chicago, IL, USA) between 10:00 AM and 12:00 PM to control diurnal variation.

**Fig 1:**
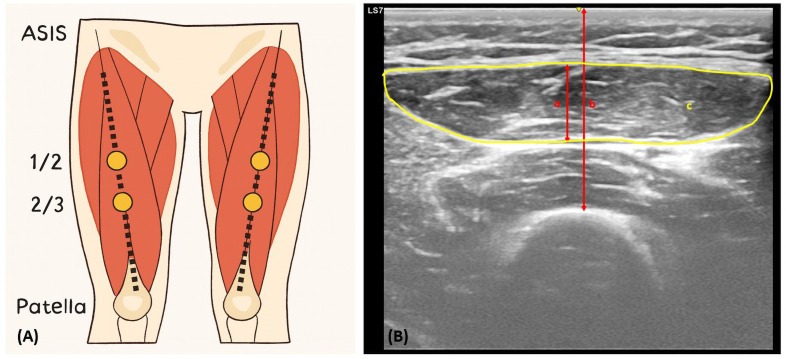
(A) Anatomical landmarks for ultrasound measurements of the rectus femoris at the midpoint (1/2) and distal third (2/3) between the anterior superior iliac spine (ASIS) and the patella. **(B)** A representative transverse ultrasound image showing the rectus femoris muscle thickness **(a)**, the total anterior thigh muscle thickness **(b)**, and the rectus femoris cross-sectional area (c, yellow contour).

Patients were scanned in the supine position with their arms and legs fully extended. Minimal probe pressure and ample contact gel were applied to avoid compressing the muscle. Measurements were obtained from two standardized anatomical landmarks on each thigh: (1) the midpoint between the anterior superior iliac spine and the superior pole of the patella and (2) the junction between the lower third and the upper two-thirds of that distance. Fig 1(A) illustrates the anatomical landmarks. The same four sites were marked bilaterally and consistently used for all serial assessments throughout the ICU stay.

To reduce operator bias, the same operator performed all serial measurements for each patient while a second trained researcher verified probe placement during each scan. All ultrasound images were independently reviewed by two investigators blinded to patient data. The following parameters were measured bilaterally: rectus femoris (RF) thickness, RF echogenicity, RFcsa, and total anterior thigh muscle thickness. Fig 1(B) shows a representative ultrasound image of the rectus femoris with delineated CSA boundaries and thickness measurements. The RFcsa was measured in the transverse plane by manually tracing the hyperechoic fascial border of the muscle. The device software automatically calculated the area. RF muscle thickness was assessed in the longitudinal plane as the perpendicular distance between the superficial and deep fasciae of the muscle. Total anterior thigh thickness was defined as the distance from the skin surface to the anterior femoral cortex, including the subcutaneous tissue, rectus femoris, and vastus intermedius muscles. RF echogenicity was quantified within the RF CSA region using built-in grayscale histogram analysis software that provided a mean brightness value as a surrogate marker of muscle quality.

### Statistical analysis

Continuous variables related to demographics and baseline clinical characteristics were summarized as means with standard deviations or medians with interquartile ranges, as appropriate. RFcsa measurements and continuous clinical outcomes were reported as medians with interquartile ranges. Categorical variables were expressed as frequencies and percentages. There was no missing data identified for RFcsa measurements, patient outcomes, demographics, or baseline clinical characteristics.

Univariable logistic regression analyses were performed to identify predictors of significant muscle wasting (≥10% reduction in RFcsa), ICU mortality, and in-hospital mortality. Variables with p-values less than 0.1 in the univariable analysis were then entered into multivariable logistic regression models using backward stepwise selection. Each model used the corresponding binary outcome as the dependent variable. Adjusted odds ratios (ORs) and 95% confidence intervals (CIs) were calculated using Wald statistics.

As this was a retrospective observational study, no formal a priori sample size calculation was performed. To ensure model stability and minimize the risk of overfitting, the number of variables in each multivariable model was limited, achieving the following event-per-variable (EPV) ratios: 41 events with two variables (EPV = 20.5) for the muscle wasting model, 18 events with three variables (EPV = 6.0) for the ICU mortality model, and 29 events with five variables (EPV = 5.8) for the in-hospital mortality model. These ratios meet accepted guidelines for reliable logistic regression estimates.

We evaluated associations between changes in RFcsa and patient characteristics or clinical outcomes using the Mann–Whitney U test for continuous variables and Fisher’s exact test for categorical variables. A two-tailed p-value of less than 0.05 was considered statistically significant. As this was a retrospective cohort study with a limited sample size, we did not routinely perform comprehensive diagnostic testing for multicollinearity, logit linearity, and interaction effects, which represents a potential limitation in model validation. All statistical analyses were performed using IBM SPSS Statistics for Windows, version 25.0 (IBM Corp., Armonk, NY, USA).

## Results

### Baseline characteristics of the study population

**Fig 2:**
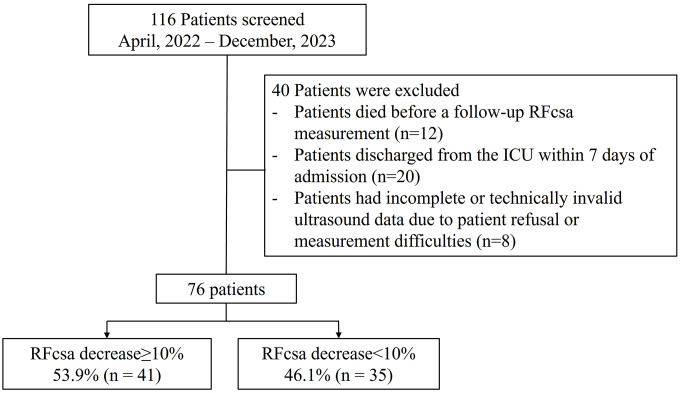
Flow diagram of patient selection: Of the 116 patients who were screened, 40 were excluded due to early death, a short ICU stay, or invalid ultrasound data. This left 76 patients for the final analysis.

A total of 116 patients were screened between April 2022 and December 2023. Of these, 40 patients were excluded: Twelve died before the follow-up RFcsa measurement, twenty were discharged from the ICU within seven days, and eight had incomplete or invalid ultrasound data. Therefore, 76 patients were included in the final analysis (Fig 2). Among them, 41 patients (53.9%) experienced a significant reduction in RFcsa (decrease of at least 10%), while 35 patients (46.1%) experienced a decrease of less than 10%.

Baseline characteristics are presented in Table 1. No significant differences were observed between the two groups in terms of age, sex, body mass index, APACHE II score, SOFA score, frailty. Among the evaluated comorbidities, solid tumors were observed in 40.0% of patients in the RFcsa < 10% group compared to 17.1% in the RFcsa ≥ 10% group (p = 0.026). Other comorbidities showed no significant differences between groups.

**Table 1:** Baseline characteristics of enrolled patients.

Characteristics	All patients (n = 76)	RFcsa decrease≥10% (n = 41)	RFcsa decrease<10% (n = 35)	P-value
Age (years)	70.5 ± 14.2	70.9 ± 13.3	70.0 ± 15.3	0.785
Male	48 (63.2%)	25 (61.0)	23 (65.7)	0.669
Body mass index (kg/m^2^)	22.5 ± 4.8	23.0 ± 5.2	21.9 ± 4.3	0.319
APACHE II score	23.8 ± 7.2	24.4 ± 6.6	23.2 ± 8.0	0.498
SOFA score	7.5 ± 3.1	7.7 ± 3.0	7.1 ± 3.2	0.388
Charlson comorbidity index	2.8 ± 2.5	2.3 ± 2.4	3.4 ± 2.6	0.057
Clinical frailty scale	4.3 ± 1.5	4.3 ± 1.5	4.2 ± 1.6	0.795
SARC-F score	3.9 ± 3.3	4.3 ± 3.5	3.5 ± 3.0	0.293
Comorbidities				
Hypertension	46 (60.5)	23 (56.1)	23 (65.7)	0.393
Diabetes	38 (50.0)	22 (53.7)	16 (45.7)	0.490
Cerebral infarction	4 (5.3)	2 (4.9)	2 (5.7)	0.871
Heart failure	16 (21.1)	6 (14.6)	10 (28.6)	0.137
Liver cirrhosis	8 (10.5)	5 (12.2)	3 (8.6)	0.608
Solid tumor	21 (27.6)	7 (17.1)	14 (40.0)	0.026
Hematologic malignancy	1 (1.3)	0 (0)	1 (2.9)	0.276
COPD	17 (22.4)	9 (22.0)	8 (22.9)	0.925
Dementia	6 (7.9)	4 (9.8)	2 (5.7)	0.515

Data are presented as mean ± standard deviation or n (%) unless otherwise noted.

RFcsa: rectus femoris cross-sectional area, APACHE: Acute Physiology And Chronic Health Evaluation, SOFA: sequential organ failure assessment, SARC-F: strength, assistance with walking, rising from a chair, climbing stairs, and falls, COPD: Chronic Obstructive Pulmonary Disease.

S1 Table summarizes the laboratory results. No statistically significant differences were found in any of the laboratory parameters between the two groups.

### ICU interventions and clinical outcomes

Table 2 presents the ICU interventions and clinical outcomes. Patients with an RFcsa decrease ≥10% had a significantly longer ICU stay than those with a decrease <10% (15.0 [11.0–22.5] vs. 10.0 [8.0–16.0] days, p = 0.001). Additionally, fewer patients in the ≥ 10% group received Level 3 or higher ICU rehabilitation than the < 10% group (4.9% vs. 20.0%, p = 0.042). No significant differences were observed between the groups regarding ICU or hospital mortality, hospital length of stay, or use of mechanical ventilation or continuous renal replacement therapy.

**Table 2:** Prognosis and interventions of patients.

Characteristics	All patients (n = 76)	RFcsa decrease≥10% (n = 41)	RFcsa decrease<10% (n = 35)	P-value
ICU LOS	12.0 (9.0–18.0)	15.0 (11.0–22.5)	10.0 (8.0–16.0)	0.001
ICU mortality	18 (23.7)	12 (29.3)	6 (17.1)	0.215
In-hospital LOS	23.0 (16.0–36.0)	30.0 (17.0–43.5)	20.0 (12.0–25.0)	0.711
In-hospital mortality	29 (38.2)	17 (41.5)	12 (34.3)	0.521
Apply of MV	69 (90.8)	39 (95.1)	30 (85.7)	0.157
Apply of CRRT	5 (6.6)	3 (7.3)	2 (5.7)	0.779
Level 3 or higher ICU rehabilitation	9 (11.8)	2 (4.9)	7 (20.0)	0.042

Data are presented as median and interquartile range or n (%) unless otherwise noted.

RFcsa: rectus femoris cross-sectional area, ICU: Intensive care unit, LOS: length of stay, MV: mechanical ventilation, CRRT: continuous renal replacement therapy.

### Changes in muscle mass during ICU stay

Table 3 displays the serial measurements of muscle mass. At baseline, the RFcsa ≥ 10% group had significantly greater RF thickness and cross-sectional area but lower echogenicity than the < 10% group. Over the 7-d observation period, the ≥ 10% group showed significantly greater reductions in RF thickness, total anterior thigh muscle thickness, and RFcsa. Changes in echogenicity did not significantly differ between the two groups.

**Table 3:** Measurements of muscle mass over time.

	All patients	RFcsa decrease≥10%	RFcsa decrease<10%	P-value
Initial (n)	76	41	35	
Rectus femoris (cm)	1.08 (0.88–1.41)	1.13 (0.93–1.47)	0.97 (0.82–1.15)	0.041
Total anterior thigh muscle thickness (cm)	3.02 (2.32–3.51)	3.24 (2.63–4.00)	2.72 (2.07–3.43)	0.055
Cross sectional area (rectus femoris, cm^2^)	4.85 (3.72–5.95)	5.41 (3.93–6.33)	4.32 (3.59–5.50)	0.020
Echogenicity, dB	45.0 (40.5–49.1)	44.6 (39.2–48.7)	46.4 (42.7–50.5)	0.038
7days (n)	76	41	35	
Rectus femoris (cm)	1.00 (0.76–1.25)	0.98 (0.80–1.31)	0.94 (0.72–1.17)	0.601
Change in Rectus Femoris Thickness (%)	−12.2 (−19.5–−0.2)	−15.6 (−23.5–−7.0)	−4.8 (−15.0–4.8)	0.009
Total anterior thigh muscle thickness (cm)	2.77 (2.10–3.25)	2.81 (2.25–3.20)	2.67 (2.03–3.30)	0.737
Change in Total Anterior Thigh Muscle Thickness (%)	−9.2 (−16.0–1.3)	−14.5 (−23.8–−6.7)	−4.0 (−12.8–8.2)	0.001
Cross sectional area (rectus femoris, cm^2^)	4.43 (3.10–5.23)	4.45 (2.81–5.15)	4.41 (3.47–5.31)	0.628
Change in Rectus Femoris Cross-Sectional Area (%)	−11.4 (−18.3–−3.4)	−17.1 (−25.6–−14.2)	−3.1 (−6.5–6.2)	<0.001
Echogenicity, dB	44.7 (40.3–49.5)	44.2 (39.6–48.2)	45.5 (41.8–50.6)	0.155
Change in Echogenicity (%)	−0.08 (−4.30–4.26)	0.27 (−2.99–6.44)	−1.33 (−6.10–3.89)	0.300

Data are presented as median and interquartile range, unless otherwise indicated.

RFcsa: rectus femoris cross-sectional area.

### Factors associated with significant muscle loss (RFcsa ≥ 10%)

Table 4 summarizes the multivariate analysis identifying factors associated with significant muscle loss, whereas S2 Table presents the univariate analysis. Participation in Level 3 or higher ICU rehabilitation was significantly associated with a reduced risk of RFcsa decrease ≥10% (OR, 0.183; 95% CI, 0.035–0.970; p = 0.046).

**Table 4:** Factors associated with RFcsa decrease ≥10% (multivariate analysis).

	Multivariate analysis		
	OR	95% CI	P-value
Charlson comorbidity index	0.821	0.675–0.998	0.048
Level 3 or higher ICU rehabilitation	0.183	0.035–0.970	0.046

RFcsa: rectus femoris cross-sectional area, OR: Odd ratio, CI: confidence interval, ICU: intensive care unit.

### Predictors of ICU mortality and in-hospital mortality

The multivariable logistic regression analysis (S3 Table) showed that higher total bilirubin (OR: 1.499; 95% CI: 1.091–2.059; p: 0.013) and lower albumin levels (OR: 0.210; 95% CI: 0.060–0.733; p: 0.014) were independently associated with an increased risk of ICU mortality. The Charlson comorbidity index showed borderline significance (p = 0.052) but did not reach statistical significance in the final model.

The following were independently associated with increased in-hospital mortality (S4 Table): the Charlson comorbidity index (OR 1.739, 95% CI 1.262–2.395, p = 0.001), low albumin (OR 0.273, 95% CI 0.079–0.937, p = 0.039), and elevated total bilirubin (OR 1.498, 95% CI 1.071–2.095, p = 0.018), and CRP levels (OR 1.098, 95% CI 1.006–1.198, p = 0.036) were independently associated with increased in-hospital mortality.

## Discussion

In this retrospective cohort study of critically ill patients in the medical ICU, we found that 53.9% experienced a ≥ 10% reduction in rectus femoris cross-sectional area (RFcsa) within the first week of admission. Patients with significant muscle wasting had longer ICU stays and were less likely to receive Level 3 ICU rehabilitation. Multivariate analysis revealed that Level ≥3 rehabilitation was independently associated with reduced muscle wasting. For mortality outcomes, elevated total bilirubin and lower albumin levels were independently associated with ICU mortality. Meanwhile, the Charlson comorbidity index, lower albumin, elevated total bilirubin, and higher CRP levels independently predicted in-hospital mortality. However, muscle wasting itself was not significantly associated with either ICU or in-hospital mortality in our cohort.

These findings align with prior studies demonstrating the early and substantial onset of muscle wasting in the ICU. Critically ill patients may lose approximately 1.75% (95% CI: −2.05 to −1.45) of RF thickness or 2.10% (95% CI: −3.17 to −1.02) of cross-sectional area per day during the first week, resulting in over 15% muscle loss [2]. Similarly, Puthucheary et al. [16] reported a 10–18% reduction in RFcsa, particularly among patients with multiorgan failure. Šostakaitė et al. [18] and Mendes et al. [19] also observed significant decreases in RF thickness using ultrasound within the first 5–7 d of ICU admission. Our results confirm that meaningful muscle loss occurs early in critical illness and support the routine application of ultrasound-based muscle monitoring in ICU settings.

The development of ICU-acquired muscle wasting is multifactorial. Contributing factors include systemic inflammation, mitochondrial dysfunction, hormonal dysregulation, and prolonged immobilization [20,21]. Elevated pro-inflammatory cytokines, such as tumor necrosis factor-alpha [22] and interleukin-6 [23,24], impaired anabolic hormone responses [25], and oxidative stress resulting from mitochondrial dysfunction [26] all exacerbate muscle degradation. Additionally, prolonged immobilization resulting from sedation and mechanical ventilation leads to disuse atrophy [27]. Early detection and interventions targeting these mechanisms are essential to prevent progressive muscle degradation.

Several risk factors for skeletal muscle loss in critically ill patients have been identified in previous studies, including age, hypoxemia, acidosis, inadequate nutrition, and greater disease severity [28,29]. Hrdy et al. [1] observed that nearly 60% of ICU patients experienced a ≥ 10% reduction in RFcsa within the first week, with age emerging as a borderline predictor. Similarly, Puthucheary et al. [16] reported RFcsa losses of up to −20.3% by day 10 in patients with four or more organ failures. Independent factors identified in their included lower bicarbonate, a reduced arterial oxygen partial pressure to fractional inspired oxygen ratio, and lower hemoglobin levels. In cohorts with SARS-CoV-2 infection, both age and SOFA scores have been associated with greater muscle loss [30]. Additionally, CRRT may contribute to muscle wasting through fluid shifts, catabolic stress, and protein loss, with previous studies suggesting associations between CRRT and skeletal muscle loss as well as increased mortality in sarcopenic patients [31,32]. In our study, comorbidity burden (CCI) showed trends toward significance; however, structured ICU rehabilitation at Level ≥3 was the only independent protective factor against muscle wasting. Interestingly, patients with solid tumors appeared to experience less muscle loss. However, nearly 40% of cancer patients were excluded due to early death or ICU discharge within seven days. This suggests that the observed association likely reflects the selection of relatively healthier survivors rather than a true protective effect. Our findings suggest that the intensity of rehabilitation may play a more decisive role in preserving skeletal muscle mass during critical illness than baseline comorbidities or acute physiological derangements.

The association of muscle wasting with adverse outcomes, including increased mortality, has been previously documented. For instance, rapid muscle loss in patients with liver cirrhosis correlated with ICU and in-hospital mortality rates of 59.3% and 77.9%, respectively [33]. Similarly, a meta-analysis reported that ICU patients with low skeletal muscle mass were more than twice as likely to die, and ICU-acquired sarcopenia was associated with increased mortality one year after critical illness [34,35]. In our study, patients with an RFcsa reduction of at least 10% had higher mortality rates; however, these differences were not statistically significant in either univariable or multivariable analyses. Lower serum albumin and elevated total bilirubin were independent predictors of ICU mortality. Higher Charlson comorbidity index, lower serum albumin, elevated total bilirubin, and higher CRP were independently associated with in-hospital mortality. These findings suggest that systemic illness severity and preexisting comorbidity burden may impact short-term outcomes more than acute muscle mass loss during the first week of the ICU stay in our relatively stable, mechanically ventilated cohort. Additionally, our relatively short follow-up period may not have captured the long-term functional consequences of muscle wasting that become apparent after ICU discharge.

From a clinical standpoint, these findings underscore the potential significance of early rehabilitation in preventing muscle wasting. Despite mounting evidence that ICU mobilization is feasible and safe [36–38], only 4.9% of patients with significant muscle loss received Level ≥3 rehabilitation in our study, compared to 20.0% of patients without significant muscle wasting. This low utilization rate represents a potential missed opportunity to intervene early and mitigate muscle loss. However, the observational nature of our study precludes definitive conclusions about the protective effect of rehabilitation. Future randomized controlled trials are needed to determine whether standardized, proactive rehabilitation protocols can effectively preserve muscle mass and improve outcomes among critically ill patients.

This study has several limitations. First, the single-center design and small sample size may limit the ability to generalize the results, though standardized protocols likely enhanced the study’s internal validity. Second, the retrospective design may have introduced selection bias regarding patients who underwent serial ultrasound measurements. Third, muscle mass was assessed using RFcsa without evaluating muscle strength or functional outcomes, such as post-ICU mobility and quality of life. Fourth, detailed nutritional data, including caloric and protein intake, were not systematically collected. This limits our ability to account for a key determinant of muscle mass in critically ill patients. Fifth, other potential confounding factors, such as inflammatory markers and fluid balance, were not systematically recorded, limiting exploration of underlying mechanisms. To minimize variability in measurements, serial ultrasound assessments were performed at standardized time points under consistent conditions, and hemodynamically unstable patients were excluded. Sixth, patients who died before the follow-up assessment were excluded. This may have inadvertently excluded the most critically ill individuals, but it likely reduced variability from extreme fluid shifts. Nearly 40% of patients with solid tumors were excluded within the first seven days due to death or ICU discharge. This may have resulted in the selection of a healthier subgroup of cancer patients, which could explain the counterintuitive finding of reduced muscle loss in this group. Seventh, the retrospective design, limited sample size, and software constraints (SPSS 25.0) prevented us from formally testing assumptions for the logistic regression models, including assessing multicollinearity, linearity of the logit, and interaction effects. This represents a methodological limitation that may affect the robustness of our multivariable analyses. Finally, the observational design precludes causal inference regarding the relationship between rehabilitation intensity and muscle preservation. Future prospective, multicenter studies with standardized rehabilitation protocols and long-term follow-up are needed to validate these findings and assess their impact on patient-centered outcomes.

## Conclusions

In this retrospective cohort study of ICU patients, more than half experienced a reduction in RFcsa of at least 10% within the first week. While this muscle loss was not associated with mortality, it was associated with longer ICU stays and reduced receipt of structured rehabilitation. Our analysis suggests that higher-level ICU rehabilitation may protect against muscle wasting; however, the observational design limits causal inference. These findings highlight the potential value of exploring early, targeted rehabilitation strategies and considering routine ultrasound monitoring to preserve muscle mass in future prospective studies.

## Supporting information

S1 Table Laboratory findings of enrolled patients. (DOCX)

S2 Table Univariate and multivariate Logistic regression analysis addressing the factors for decreases in RFcsa ≥ 10%. (DOCX)

S3 Table Univariate and multivariate Logistic analysis addressing the factors for ICU mortality. (DOCX)

S4 Table Univariate and multivariate Logistic analysis addressing the factors for in-hospital mortality. (DOCX)
