# Correction: Early skeletal muscle loss and clinical outcomes in critically ill patients in the medical intensive care unit: A retrospective cohort study

**DOI:** 10.1371/journal.pone.0343790

**Published:** 2026-02-25

**Authors:** The PLOS One Staff

## Notice of Republication

This article was republished on February 6, 2026, to correct an error in the sixth author’s name. The publisher apologises for this error. The correct name is: Song I Lee. The correct citation is: Kim S, Kang DH, Kim D, Ahn S, Lee MR, Lee SI (2025) Early skeletal muscle loss and clinical outcomes in critically ill patients in the medical intensive care unit: A retrospective cohort study. PLoS One 20(12): e0338315. https://doi.org/10.1371/journal.pone.0338315

## Supporting information

S1 File Originally published, uncorrected article. (PDF)

S2 File Republished, corrected article. (PDF)
