# Supplementary material for: Early skeletal muscle loss and clinical outcomes in critically ill patients in the medical intensive care unit: A retrospective cohort study
Source: PLoS One. 2025 Dec 18;20(12):e0338315. doi: 10.1371/journal.pone.0338315 (PMC12714274; doi:10.1371/journal.pone.0338315)
Supplement: S1 Table — (DOCX) [file pone.0338315.s001.docx]

**Clinical significance of muscle wasting in medical intensive care patients: A Retrospective Cohort Study**
Supporting information

S1 Table. Laboratory findings of enrolled patients

| Characteristics | All patients  (n = 76) | RFcsa decrease≥10% (n = 41) | RFcsa decrease<10% (n = 35) | P-value |
| --- | --- | --- | --- | --- |
| White blood cell, ×10^3^/uL | 13.4 ± 10.1 | 13.1 ± 8.5 | 13.8 ± 11.9 | 0.745 |
| Hemoglobin, g/dL | 11.3 (9.6 – 12.4) | 11.1 (9.7 – 12.2) | 11.4 (9.3 – 13.1) | 0.391 |
| Platelet, ×10^3^/uL | 210.6 ± 134.1 | 213.5 ± 143.9 | 207.3 ± 123.7 | 0.842 |
| Total bilirubin, mg/dL | 0.79 (0.56 – 1.43) | 0.68 (0.54 – 1.48) | 0.94 (0.60 – 1.44) | 0.083 |
| Albumin, g/dL | 3.1 ± 0.6 | 3.0 ± 0.6 | 3.2 ± 0.6 | 0.187 |
| AST, U/L | 38 (23 – 61) | 30 (23 – 60) | 40 (23 – 71) | 0.382 |
| ALT, U/L | 23 (15 – 42) | 24 (15 – 39) | 22 (15 – 49) | 0.467 |
| BUN, mg/dL | 33.0 ± 29.0 | 31.4 ± 20.1 | 34.9 ± 37.0 | 0.598 |
| Creatinine, mg/dL | 0.86 (0.61 – 1.93) | 1.12 (0.62 – 1.89) | 0.74 (0.61 – 2.02) | 0.431 |
| Na, mEq/L | 137.4 ± 6.3 | 137.5 ± 5.6 | 137.2 ± 7.0 | 0.825 |
| K, mEq/L | 4.3 ± 1.0 | 4.2 ± 0.9 | 4.4 ± 1.1 | 0.270 |
| Cl, mEq/L | 102.7 ± 7.1 | 101.9 ± 6.7 | 103.7 ± 7.4 | 0.264 |
| CRP, ng/mL | 7.9 (3.7 – 8.0) | 8.0 (4.4 – 8.0) | 7.4 (2.5 – 8.0) | 0.323 |
| Procalcitonin, ng/mL | 0.88 (0.11 – 5.24) | 1.80 (0.11 – 8.49) | 0.49 (0.11 – 4.23) | 0.382 |
| PT, INR | 1.35 ± 0.42 | 1.37 ± 0.47 | 1.32 ± 0.37 | 0.645 |
| aPTT. Sec | 33.9 ± 11.8 | 32.7 ± 6.2 | 35.4 ± 16.2 | 0.333 |
| Lactic acid, mEq/L | 2.15 (1.43 – 3.80) | 2.30 (1.80 – 3.70) | 1.70 (1.30 – 3.80) | 0.492 |

Data are presented as mean ± standard deviation or median and interquartile range, unless otherwise indicated.

RFcsa: rectus femoris cross-sectional area, AST: Aspartate transaminase, ALT: Alanine transaminase, BUN: Blood urea nitrogen, Na: Sodium, K: potassium, Cl: Chloride, CRP: C-reactive protein, PT: Prothrombin time, aPTT: Activated partial thromboplastin time
