# Supplementary material for: Early skeletal muscle loss and clinical outcomes in critically ill patients in the medical intensive care unit: A retrospective cohort study
Source: PLoS One. 2025 Dec 18;20(12):e0338315. doi: 10.1371/journal.pone.0338315 (PMC12714274; doi:10.1371/journal.pone.0338315)
Supplement: S2 Table — (DOCX) [file pone.0338315.s002.docx]

**Clinical significance of muscle wasting in medical intensive care patients: A Retrospective Cohort Study**
Supporting information

S2 Table. Univariate and multivariate Logistic regression analysis addressing the factors for decreases in RFcsa≥10%

|  | Univariate analysis | | | Multivariate analysis | | |
| --- | --- | --- | --- | --- | --- | --- |
|  | OR | 95% CI | P-value | OR | 95% CI | P-value |
| Age, yr | 1.005 | 0.973 – 1.037 | 0.782 |  |  |  |
| Male | 0.815 | 0.319 – 2.084 | 0.670 |  |  |  |
| Body mass index, kg/m^2^ | 1.051 | 0.954 – 1.158 | 0.316 |  |  |  |
| APACHE II score | 1.023 | 0.959 – 1.090 | 0.493 |  |  |  |
| SOFA score | 1.068 | 0.921 – 1.240 | 0.384 |  |  |  |
| Charlson comorbidity index | 0.833 | 0.687 – 1.010 | 0.064 | 0.821 | 0.675 – 0.998 | 0.048 |
| Clinical frailty scale | 1.041 | 0.774 – 1.400 | 0.791 |  |  |  |
| SARC-F score | 1.078 | 0.938 – 1.238 | 0.288 |  |  |  |
| Laboratory findings |  |  |  |  |  |  |
| White blood cell, ×10^3^/uL | 0.992 | 0.949 – 1.038 | 0.742 |  |  |  |
| Hemoglobin, g/dL | 1.025 | 0.949 – 1.107 | 0.527 |  |  |  |
| Platelet, ×10^3^/uL | 1.000 | 0.997 – 1.004 | 0.839 |  |  |  |
| Total bilirubin, mg/dL | 1.247 | 0.920 – 1.689 | 0.155 |  |  |  |
| Albumin, g/dL | 0.588 | 0.267 – 1.294 | 0.187 |  |  |  |
| Creatinine, mg/dL | 0.911 | 0.761 – 1.137 | 0.411 |  |  |  |
| CRP, ng/mL | 1.034 | 0.967 – 1.106 | 0.326 |  |  |  |
| Lactate, mmol/L | 1.047 | 0.919 – 1.193 | 0.490 |  |  |  |
| Interventions in the ICU |  |  |  |  |  |  |
| Invasive mechanical ventilation | 3.250 | 0.589 – 17.924 | 0.176 |  |  |  |
| Continuous renal replacement therapy | 1.303 | 0.205 – 8.276 | 0.779 |  |  |  |
| Level 3 or higher ICU rehabilitation | 0.205 | 0.040 – 1.063 | 0.059 | 0.183 | 0.035 – 0.970 | 0.046 |

OR, odd ratio; CI, confidence interval; APACHE, Acute physiology and chronic health evaluation; SOFA, sequential organ failure assessment; SARC-F, strength, assistance with walking, rising from a chair, climbing stairs, and falls; CRP, c-reactive protein; ICU, intensive care unit
