# Supplementary material for: Early skeletal muscle loss and clinical outcomes in critically ill patients in the medical intensive care unit: A retrospective cohort study
Source: PLoS One. 2025 Dec 18;20(12):e0338315. doi: 10.1371/journal.pone.0338315 (PMC12714274; doi:10.1371/journal.pone.0338315)
Supplement: S3 Table — (DOCX) [file pone.0338315.s003.docx]

**Clinical significance of muscle wasting in medical intensive care patients: A Retrospective Cohort Study**
Supporting information

S3 Table. Univariate and multivariate Logistic analysis addressing the factors for ICU mortality

|  | Univariate analysis | | | Multivariate analysis | | |
| --- | --- | --- | --- | --- | --- | --- |
|  | OR | 95% CI | P-value | OR | 95% CI | P-value |
| Age, yr | 1.000 | 0.963 – 1.038 | 0.996 |  |  |  |
| Body mass index, kg/m^2^ | 0.984 | 0.879 – 1.101 | 0.776 |  |  |  |
| APACHE II score | 1.054 | 0.979 – 1.135 | 0.162 |  |  |  |
| SOFA score | 1.126 | 0.941 – 1.347 | 0.194 |  |  |  |
| Charlson comorbidity index | 1.241 | 1.011 – 1.524 | 0.039 | 1.263 | 0.998 – 1.597 | 0.052 |
| Clinical frailty scale | 1.116 | 0.789 – 1.578 | 0.536 |  |  |  |
| SARC-F score | 1.010 | 0.860 – 1.185 | 0.907 |  |  |  |
| Laboratory findings |  |  |  |  |  |  |
| White blood cell, ×10^3^/uL | 1.022 | 0.974 – 1.073 | 0.374 |  |  |  |
| Platelet, ×10^3^/uL | 0.997 | 0.993 – 1.002 | 0.253 |  |  |  |
| Total bilirubin, mg/dL | 1.391 | 1.044 - 1.853 | 0.024 | 1.499 | 1.091 – 2.059 | 0.013 |
| Albumin, g/dL | 0.293 | 0.098 – 0.872 | 0.027 | 0.210 | 0.060 – 0.733 | 0.014 |
| Creatinine, mg/dL | 0.755 | 0.487 – 1.172 | 0.210 |  |  |  |
| CRP, ng/mL | 1.056 | 0.988 – 1.129 | 0.111 |  |  |  |
| Lactate, mmol/L | 0.988 | 0.850 – 1.147 | 0.871 |  |  |  |
| RFcsa decrease≥10% | 1.369 | 0.465 – 4.026 | 0.569 |  |  |  |

OR, odd ratio; CI, confidence interval; APACHE, Acute physiology and chronic health evaluation; SOFA, sequential organ failure assessment; SARC-F, strength, assistance with walking, rising from a chair, climbing stairs, and falls; CRP, c-reactive protein; ICU, intensive care unit
