# Supplementary material for: Early skeletal muscle loss and clinical outcomes in critically ill patients in the medical intensive care unit: A retrospective cohort study
Source: PLoS One. 2025 Dec 18;20(12):e0338315. doi: 10.1371/journal.pone.0338315 (PMC12714274; doi:10.1371/journal.pone.0338315)
Supplement: S4 Table — (DOCX) [file pone.0338315.s004.docx]

**Clinical significance of muscle wasting in medical intensive care patients: A Retrospective Cohort Study**
Supporting information

S4 Table. Univariate and multivariate Logistic analysis addressing the factors for in-hospital mortality

|  | Univariate analysis | | | Multivariate analysis | | |
| --- | --- | --- | --- | --- | --- | --- |
|  | OR | 95% CI | P-value | OR | 95% CI | P-value |
| Age, yr | 0.996 | 0.964 – 1.029 | 0.790 |  |  |  |
| Body mass index, kg/m^2^ | 0.980 | 0.888 – 1.081 | 0.686 |  |  |  |
| APACHE II score | 1.044 | 0.978 – 1.115 | 0.199 |  |  |  |
| SOFA score | 1.153 | 0.982 – 1.353 | 0.082 | 1.142 | 0.919 – 1.420 | 0.231 |
| Charlson comorbidity index | 1.447 | 1.154 – 1.815 | 0.001 | 1.739 | 1.262 – 2.395 | 0.001 |
| Clinical frailty sc3~5ale | 1.268 | 0.929 – 1.730 | 0.135 |  |  |  |
| SARC-F score | 1.049 | 0.911 – 1.208 | 0.506 |  |  |  |
| Laboratory findings |  |  |  |  |  |  |
| White blood cell, ×10^3^/uL | 1.001 | 0.956 – 1.048 | 0.979 |  |  |  |
| Platelet, ×10^3^/uL | 0.999 | 0.996 – 1.003 | 0.657 |  |  |  |
| Total bilirubin, mg/dL | 1.350 | 0.991 – 1.839 | 0.057 | 1.498 | 1.071 – 2.095 | 0.018 |
| Albumin, g/dL | 0.443 | 0.186 – 1.051 | 0.065 | 0.273 | 0.079 – 0.937 | 0.039 |
| Creatinine, mg/dL | 0.750 | 0.531 – 1.060 | 0.103 |  |  |  |
| CRP, ng/mL | 1.062 | 0.991 – 1.138 | 0.090 | 1.098 | 1.006 – 1.198 | 0.036 |
| Lactate, mmol/L | 0.983 | 0.863 – 1.119 | 0.792 |  |  |  |
| RFcsa decrease≥10% | 1.358 | 0.533 – 3.456 | 0.521 |  |  |  |

OR, odd ratio; CI, confidence interval; APACHE, Acute physiology and chronic health evaluation; SOFA, sequential organ failure assessment; SARC-F, strength, assistance with walking, rising from a chair, climbing stairs, and falls; CRP, c-reactive protein; ICU, intensive care unit
